# iTRAQ-based proteomics analysis on insomnia rats treated with Mongolian medical warm acupuncture

**DOI:** 10.1042/BSR20191517

**Published:** 2020-05-07

**Authors:** Yanan Xu, Xian Li, Duriwa Man, Xiulan Su, Gula A

**Affiliations:** 1 Clinical Medicine Research Center of Affiliated Hospital, Inner Mongolia Medical University, No.1 Tongdao North Street, Hohhot, Inner Mongolia 010050, China; 2 Inner Mongolia Medical University, No.1 Tongdao North Street, Hohhot, Inner Mongolia 010050, China

**Keywords:** insomnia, iTRAQ, proteomics, Traditional Mongolian medicine, warm acupuncture

## Abstract

Objective: To explore the proteomic changes in the hypothalamus of rats treated with Mongolian medical warm acupuncture for insomnia therapy based proteomics. Method: We used an iTRAQ-based quantitative proteomic approach to identify proteins that potential molecular mechanisms involved in the treatment of insomnia by Mongolian medical warm acupuncture. Result: In total, 7477 proteins were identified, of which 36 proteins showed increased levels and 45 proteins showed decreased levels in insomnia model group (M) compared with healthy control group (C), 72 proteins showed increased levels and 44 proteins showed decreased levels from the warm acupuncture treated insomnia group (W) compared with healthy controls (C), 28 proteins showed increased levels and 17 proteins showed decreased levels from the warm acupuncture-treated insomnia group (W) compared with insomnia model group (M). Compared with healthy control groups, warm acupuncture-treated insomnia group showed obvious recovered. Bioinformatics analysis indicated that up-regulation of neuroactive ligand–receptor interaction and oxytocin signaling was the most significantly elevated regulate process of Mongolian medical warm acupuncture treatment for insomnia. Proteins showed that increased/decreased expression in the warm acupuncture-treated insomnia group included Prolargin (PRELP), NMDA receptor synaptonuclear-signaling and neuronal migration factor (NSMF), Transmembrane protein 41B (TMEM41B) and Microtubule-associated protein 1B (MAP1B) to adjust insomnia. Conclusion: A combination of findings in the present study suggest that warm acupuncture treatment is efficacious in improving sleep by regulating the protein expression process in an experimental rat model and may be of potential benefit in treating insomnia patients with the added advantage with no adverse effects.

## Introduction

According to a report by the World Health Organization, about 1/3 of the world’s population suffers from sleep disorders, and the percentage in the Chinese population, which is 27%, is significantly higher than the average levels worldwide [1]. Insomnia also leads to diseases such as hypertension, low immune function and diabetes. The current understanding of clinical science on insomnia is limited, and hypnotics are primarily used for treatment. However, drug treatment is usually accompanied by many side effects, and with poor long-term efficacy [2,3].

Mongolian medical warm acupuncture therapy is a traditional Mongolian therapy that prevents and treats diseases by stimulating specific or other sites (acupoints) of human body, using special silver acupuncture needles, combined with heat stimulation. It functions by unclogging meridians, regulating blood circulation, which results in strengthening the immunity system, and preventing and/or treating diseases. Mongolian medicine annals documentation and modern clinical studies have proven the effectiveness of Mongolian medical warm acupuncture in treating insomnia, and have shown that it is safer than hypnotics as it does not possess any side effects. However, the specific mechanism warm acupuncture-treated insomnia of remains unclear [4,5].

In the post-genome era, the important role of proteins, as executors of many biological functions, has become apparent in life sciences [6]. High-throughput analysis of protein expression levels, modifications, and protein–protein interactions in organisms, tissues, and cells, can provide valuable information that can be potentially used to improve disease diagnosis and prognosis [7]. Studies of individual proteins using traditional biochemical techniques require huge workload that is almost impossible to achieve when examining the whole proteome of an organism, hence making the identification of new pathogenesis mechanisms more difficult. Therefore, in order to study the molecular mechanism of Mongolian medical warm acupuncture in the treatment of insomnia, in the present study we employed proteomics technologies as a new systems biology tool for the first time. Previous studies have shown that iTRAQ, a new quantitative comparative proteomics technique, possesses outstanding advantages when compared with research strategies based on 2D-gel electrophoresis and mass spectrometry analysis [8,9].

Using iTRAQ-based proteomics, the present study explored the proteomic changes in the hypothalamus of rats treated with Mongolian medical warm acupuncture for insomnia therapy. The animals were divided into three groups: the normal control group (C), the insomnia model group (M) and the warm acupuncture-treated insomnia group (W). Hypothalamus samples from each group were enzymatically processed and chemically labeled with distinct iTRAQ labeling agents, and proteins with differential levels in the treatment group were identified through multidimensional liquid chromatography-tandem mass spectrometry analysis. The results obtained by systems analysis, and the potential molecular mechanisms involved in the treatment of insomnia by Mongolian medical warm acupuncture are discussed.

## Materials and methods

### Insomnia rat model construction

We purchased 30 healthy male Sprague-Dawley rats aged 8–10 weeks and weighing 220 ± 10 g from the Department of Laboratory Animals of Peking University Health Science Center. The protocol was approved by the Laboratory Animal Welfare Committee. The animal experiments conformed the U.S. National Institutes of Health guidelines and the guidelines from Directive 2010/63/EU of the European parliament on the protection of animals used for scientific purposes.

Animal experiments were completed at the animal experiment center of Inner Mongolia medical university. All animals were maintained under controlled conditions (21–22°C; 55–65% humidity; 12-h light, 12-h dark cycle) with free access to rodent soy-free food and water. The animals were acclimated to their new circumstances for one week. Then, the animals were randomly divided into three groups (*n*=10/group): healthy controls (C), insomnia model group (M) and the warm acupuncture-treated insomnia group (W).

Rats in the M and W groups use peritoneal injection of p-chlorophenylalanine (300 mg/ kg) to construct the insomnia rat model, and the dosage was adjusted with the increase in body weight. The concentrations have been described previously. In the warm acupuncture group, the rats were adaptively raised for 7 days and intraperitoneally injected with normal saline (0.l ml/kg) between 8:30 and 9:00 a.m. Each day for two consecutive days from the eight day. Starting from the 10th day. Acupuncture treatment was performed at 08:00 every morning. Stainless steel acupuncture needles (0.35 mm × 20 mm, supplied by Inner Mongolia Yuanyang Traditional Chinese and Mongolian Science and Technology Development LLC) were used. Depth of needling at each point was 5 mm. The Dinghui Acupoint, Heyi Acupoint, and Xin Acupoint of each rat were stimulated with warm acupuncture for 15 min each time (Mongolian Model MY-I electric heating needle warmers) at ∼40°C (be careful not to burn the needling site) once each day for seven consecutive days [5,10].

### Sample collection

All rats were killed at the anestrus period following anesthesia under 1% sodium pentobarbital (40 mg/kg intraperitoneal injection). Collected rats hypothalamic tissue. Finally, rats were euthanized by an excessive dose of 1% sodium pentobarbital (intraperitoneal injection).

### Protein preparation

The hypothalamus of all rat groups were harvested, SDT buffer was added to the sample, and then transferred into 2 ml centrifuge tubes, prefilled with appropriate amount of quartz sand and an additional 1/4 inch of ceramic bead. Following, the samples were homogenized in a MP tissue homogenizer (24×2, 6.0 M/S, 60 s, twice), ultrasonificated (80 W, 10-s ultrasound, 15-s interval, 10 cycles), and then incubated in boiling water for 15 min. After centrifugation at 14,000 ***g*** for 40 min, supernatants were filtered through a 0.22-µm filter. Protein concentrations were determined using the BCA method. The sample was stored a −80°C [11].

### SDS-PAGE separation

For each sample, 20 μg of proteins were mixed with 5× loading buffer, boiled for 5 min, and then separated by 12.5% SDS-PAGE (constant current of 14 mA, 90 min). Protein bands were visualized using Coomassie Blue staining.

### Filter aided sample preparation (FASP) method

For each group, 200 μg of proteins for each sample were incorporated into 30 μl SDT buffer (4% SDS, 100 mM DTT, 150 mM Tris-HCl pH 8.0). Following, the mixtures were boiled for 5 min, and then cooled down to room temperature. Next, 200 ml of UA buffer was added, mixed up, and transferred into 10 kDa ultrafiltration tubes and centrifuged at 14,000 ***g*** for 15 min, the filtrate was discarded (this step was repeated once). About 100 μl of iodoacetamide (IAA) buffer (100 mM IAA in UA) was added, then vortexed at 600 rpm for 1 min, and the mixture reacted for 30 min at room temperature, under dark conditions; then centrifuged at 14,000 ***g*** for 15 min. About 100 μl of UA buffer was added and the sample was centrifuged at 14,000 ***g*** for 15 min; the process was repeated twice. About 100 μl of 10 times diluted dissolution medium was added, and the sample was centrifuged at 14,000 ***g*** for 15 min; this step was repeated twice. Following, 40 μl of trypsin buffer (4 μg trypsin in 40 μl dissolution buffer) was added, mixed by vortexing at 600 rpm for 1 min, and then incubated at 37°C for 16–18 h. The sample column was transferred into a new collection tube, and centrifuged at 14,000 ***g*** for 15 min; then the sample was eluted by adding 40 μl of 10 times diluted dissolution buffer, followed by centrifugation at 14,000 ***g*** for 15 min. Peptides were desalinated using a C18 cartridge, and after lyophilization were re-suspended in 40 μl of dissolution buffer. Peptide concentration was estimated by measuring absorbance at OD280 [12].

### iTRAQ labeling

About 100 μg peptide mixture of each sample was labeled using iTRAQ reagent according to the manufacturer’s instructions (Applied Biosystems). The experiment was carried out three times.

### Peptide separation using strong cation exchange (SCX) chromatography

Labeled peptides from each group were mixed and separated using the AKTA Purifier 100. About 10 mM KH_2_PO_4_ with 25% ACN (pH 3.0) was used as buffer A, and 10 mM KH_2_PO_4_, 500 mM KCl with 25% ACN (pH 3.0) was used as buffer B. The chromatographic column was equilibrated with buffer A, and samples were loaded into the column through a sample injector with flow rate adjusted at 1 ml/min. The gradient was set as follows: linear gradient of 0–8% buffer B for 0–22 min; linear gradient of 8–52% buffer B for 22–47 min; linear gradient of 52–100% buffer B for 47–50 min; after 58 min buffer B concentration was set to 0%. During elution, the absorbance at 214 nm was recorded, and eluted solutions were collected every 1 min; totally, 30 eluted solutions were collected. After lyophilization the samples were desalinated using a C18 cartridge.

### Mass spectrometry analysis

#### HPLC

Samples were analyzed using nano LC-MS/MS. Peptide mixtures were loaded with buffer A (0.1% formic acid) on a reversed-phase column, and then eluted under a linear gradient of buffer B (84% acetonitrile and 0.1% formic acid). The flow rate was adjusted at 300 nl/min.

#### LC-MS/MS analysis

After chromatography separation, samples were analyzed on the Q-Exactive mass spectrometer. Each sample required 60 min of analysis, and radical cations were used as fragment ions; the scan range was set at 300–1800 *m/z*. Resolution of the primary mass spectrometry was 70,000 at 200 *m/z*, and the AGC target was 3e6; the primary Maximum IT was 10 ms, and the number of scan ranges was 1; dynamic exclusion was set at 40.0 s. The mass electron ratio of peptides and fractions were collected as follows: 10 fraction spectra were collected (MS2 scan) after each full scan; the MS2 activation type was HCD, and the isolation window was 2 *m/z*. The resolution of secondary mass spectrometry was 17,500 at 200 *m/z*; microscans: 1; the secondary maximum IT was 60 ms, normalized collision energy was 30eV, and underfill was 0.1%.

### Data analysis

MASCOT is considered the golden standard in the proteomics qualitative analysis, thus, Mascot 2.2 was used for database searching in this study. The Uniprot protein Rattus norvegicus database (35,838 sequences) was used for search. Caramidomethylation of cysteines, iTRAQ labeling at the N-term and the lysine side chain amion groups were set as fixed modification. The oxidation of methionine and iTRAQ 4 plex(Y) were set as variable modification. The false discovery rate (FDR) for peptide was set to 1%. The minimum peptide length was set to 6. Enzyme specificity was set to be Trypsin and up to two missed cleavages per peptide were allowed. Mass tolerance for precursor ions was set to 20 ppm and for fragment ions at 0.1Da.

### Gene Ontology (GO) analysis

The process of GO annotations was performed on the targeted protein clusters using Blast2GO, and was divided into four steps: sequence alignment, extraction of GO annotations, GO annotation and supplementary annotation [13]. First, the targeted protein clusters were aligned with the corresponding protein sequences in the database, using the localization sequence alignment tool NCBI BLAST+ (ncbi-blast-2.2.28+-win32.exe), and then the first 10 aligned sequences with *E*-value ≤ 1e-3 were saved for further analysis. Following, the GO annotations that were associated with the proteins with high similarities with the target protein clusters or with homologous proteins, which had been blasted by the Blast2GO Command Line (dataset version: go_201608.obo, www.geneontology.org), were extracted. During annotation, Blast2GO Command Line annotated the extracted GO annotations to target proteins through comprehensive examination of the sequence similarities, GO annotation resource reliabilities, and the existence of circle plots between target and aligned proteins. After annotation, to further improve the annotation efficiency, conserved motifs in EBI database that matched with target proteins were scanned using InterProScan [14], and the motif-related functional information were annotated to target protein sequences. ANNEX was used to further supplement the annotated information, and linkages between different GO types were established to improve the annotation accuracy.

### KEGG pathway analysis

In the KEGG pathway database, KO (KEGG Orthology) terms were used for classification of genes and their product. Orthologous genes and their products were classified in the same pathway group, and were annotated with the same KO (or K) term. During KEGG pathway analysis of target protein clusters, first KEGG genes data were mapped, using KAAS (KEGG Automatic Annotation Server) [15], to KO terms in order to classify the target protein sequences, and then information of pathways containing these target proteins was automatically extracted according to KO classification.

### Functional enrichment analysis

While conducting enrichment analysis of the GO annotations or KEGG pathway annotations of target protein clusters, Fisher’s exact test was used to compare the distribution of each GO classification or KEGG pathway between target protein clusters and total protein clusters, and for evaluating the significance of enrichment of the corresponding GO term or KEGG pathway.

### Cluster analysis

Protein expression related data were analyzed using hierarchical cluster analysis. For hierarchical cluster analysis, clusters were selected and hot-plots were drafted using Cluster3.0 software (http://bonsai.hgc.jp/∼mdehoon/software/cluster/software.htm), and similarity definition was calculated using Java Treeview (http://jtreeview.sourceforge.net) with Euclidean distance and average linkage hierarchical clustering.

## Results

**Figure 1:**
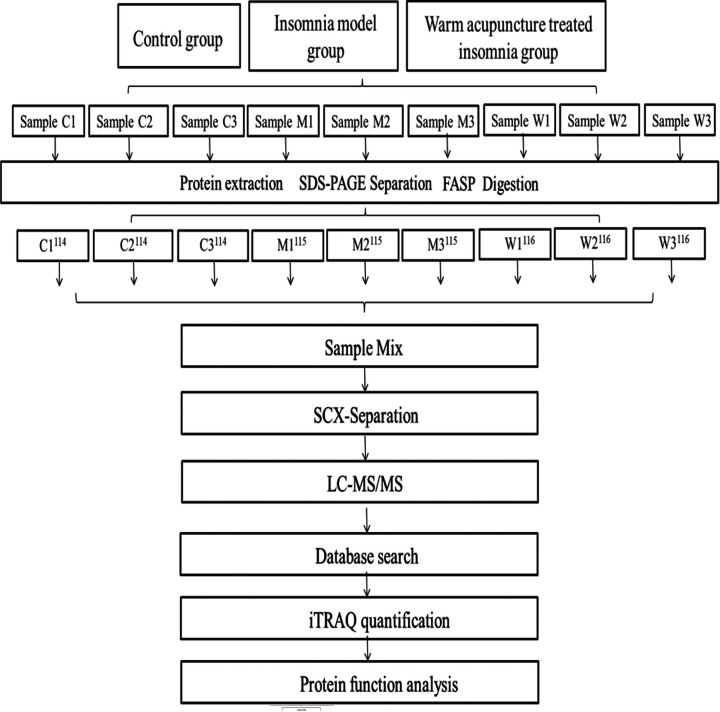
Experimental design for the quantitative proteomics analysis The experiment is divided into three groups (C, M and W). Extracted proteins were prepared via FASP and labeled with iTRAQ regents. The labeled peptides were separated by SCX chromatography and fractions were analyzed by reversed phase LC-MS/MS, all data were analyzed by bioinformatics tools from different aspects.

Differentially expressed rats hypothalamus proteins among C, M and W groups were identified and analyzed in this study using LC–MS/MS. The entire experimental process is shown in Figure 1.

### LC–MS/MS

The results indicated that a total of 7477 corresponding proteins (Supplementary Table S1) and fold change of differentially expressed proteins among C, W and M groups were determined based on the intergroup ratio in iTRAQ reports. Proteins that conformed to the following screening criteria were deemed as differentially expressed: expression fold change > 1.2 (up- or down-regulation) and *P*-value<0.05, while the protein difference multiple <0.8 and *P*<0.05 are the difference significant proteins. Comparisons between M/C, W/C and W/M groups were carried out, with altogether 81 (including 36 up- and 45 down-regulated), 116 (72 up- and 44 down-regulated) and 45 (28 up- and 17 down-regulated) differentially expressed proteins identified, respectively (Supplementary Table S2).

Optimized screening criteria, for those proteins where in differences could be seen between W/M and W/C groups, with no difference being seen M/C groups, or those differences showing opposite variation trend. The identified proteins were further screened to yield a total of 45 optimized differentially expressed proteins (Table 1).

**Table 1:** Optimized differentially expressed proteins between groups

Protein ID	Protein	Fold change	*P-*value
A0A096MK16	Protein Arap2 (Fragment)	1.236180578	0.000882046
F1M1E4	Protein Pnkd	1.203434233	0.002100419
B1H223	Down syndrome critical region gene 3	1.221180934	0.002523703
P02680	Fibrinogen gamma chain	1.233337549	0.002750139
P14480	Fibrinogen beta chain	1.205154782	0.006176793
Q6IFW6	Keratin, type I cytoskeletal 10	1.586636239	0.006670983
A8IRI3	Glucocorticoid receptor	1.374519231	0.007279957
A0A0G2KAQ8	Peroxisomal membrane protein 2	1.253174259	0.007771405
Q62649	Neuronatin	1.224901775	0.012138775
A0A0G2JWX4	Keratin, type II cytoskeletal 2 epidermal	1.56566129	0.012393709
A0A0G2K4U7	Protein Mtus2	1.232703408	0.01391488
D4A1U8	Protein RGD1311744	1.277139639	0.014144665
P24090	Alpha-2-HS-glycoprotein	1.296358037	0.01441777
F1LYI7	Protein Tmem256	1.207978689	0.015866603
D3ZA53	Protein Pnmal1	1.421893598	0.016281749
Q9EQP5	Prolargin	1.273012448	0.017232379
M0RA79	Protein LOC691828	1.273971816	0.019054036
Q95571	RT1.A(U) alpha chain (Fragment)	1.266842428	0.019175113
M0R6Y8	Phosphoglycerate kinase	1.22539767	0.024665028
Q9ESM0	Inositol hexakisphosphate kinase 1	1.274174463	0.025457038
D4AAJ2	NMDA receptor synaptonuclear-signaling and neuronal migration factor	1.227341863	0.029753527
O08562	Sodium channel protein type 9 subunit alpha	1.421454082	0.034185457
Q5FVN2	Transmembrane protein 41B	1.32306624	0.036331955
D4A8N1	Protein Dpm1	1.253915809	0.036970623
O88752	Epsilon 1 globin	1.533583884	0.040836379
D4A4T6	Protein Rint1	1.307495336	0.042824768
P17209	Myosin light chain 4	1.254133152	0.048826237
D3ZBP4	Protein-methionine sulfoxide oxidase MICAL1	1.220699776	0.049482287
O55145	Fractalkine	0.821730332	0.002073597
D3ZC82	Protein Nufip2	0.780885927	0.003504731
D3ZIC4	Protein phosphatase 1 regulatory subunit	0.797201943	0.006803938
Q3KR51	HMP19 protein, isoform CRA_a	0.813904866	0.009560059
P58405	Striatin-3	0.811684263	0.015950492
Q4KLX9	Protein Ccdc163	0.716197358	0.016416855
D3ZWP8	Protein Lrrc58	0.756999583	0.017218939
Q5UAJ6	Cytochrome *c* oxidase subunit 2	0.735583341	0.021848769
A0A0G2K7Z3	Acyl-coenzyme A thioesterase 1	0.824303275	0.024433777
P15205	Microtubule-associated protein 1B	0.47872137	0.02960253
F1LW77	Protein Rab33b	0.811272188	0.032017895
A0A0G2JW88	Microtubule-associated protein	0.82499354	0.033513215
F1LWM1	Protein Ssh1	0.765742084	0.037036806
Q6AXT8	Splicing factor 3A subunit 2	0.790258668	0.045489055
A0A140UHW6	Protein Trmt1	0.792470864	0.046071567
A1L1L5	Ccnk protein	0.819841333	0.046441222
A0A0G2K1W9	Lactate dehydrogenase D, isoform CRA_d	0.679338388	0.048325937

The proteins above have shown differences between W/M and W/C groups groups.

### Cluster analysis

**Figure 2:**
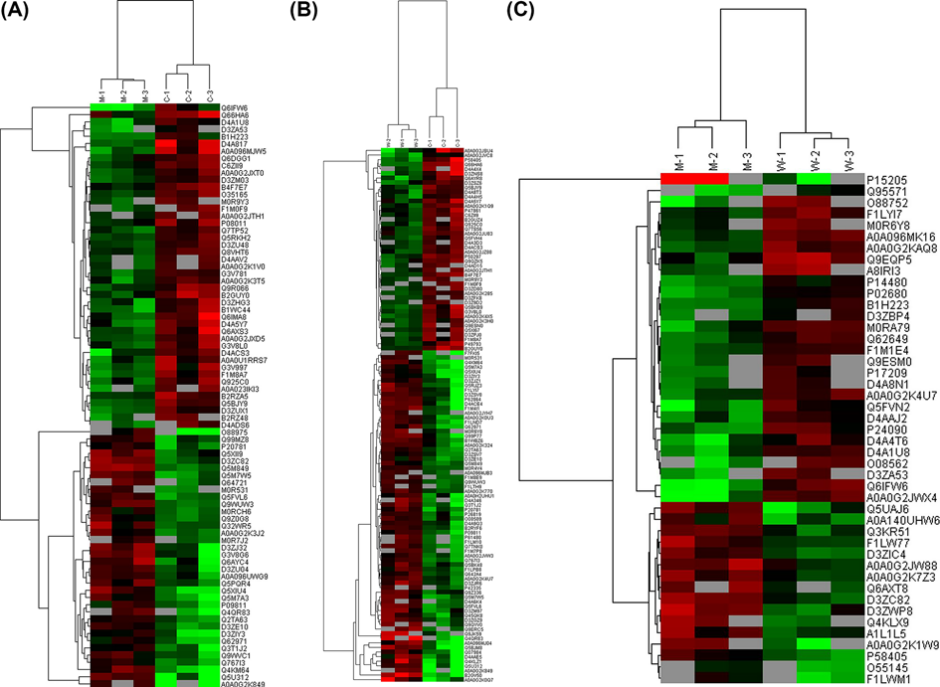
Cluster analysis of differential level proteins between (A) M versus C, (B) W versus C and (C) W versus M Colors indicate the differential protein levels, which increase successively from green to dark red. Increased levels of proteins are indicated in red, and decreased levels are marked in green.

First, cluster analysis was conducted (Figure 2). Hierarchical clustering results were expressed as a tree heat map, with red representing up-regulation and green indicating down-regulation. As determined through horizontal comparison, samples could be classified into three categories: C1-C3, M1-M3and W1-W3. Such vertical comparison indicated that proteins could be classified into two categories with opposite directional variation, which displayed the expression patterns of differentially expressed proteins in three groups, demonstrating the rationality of the selected differentially expressed proteins. The cluster analysis process thus supported that the differentially expressed proteins screened out in this experiment were reasonable and accurate.

### GO functional annotation and analysis

**Figure 3:**
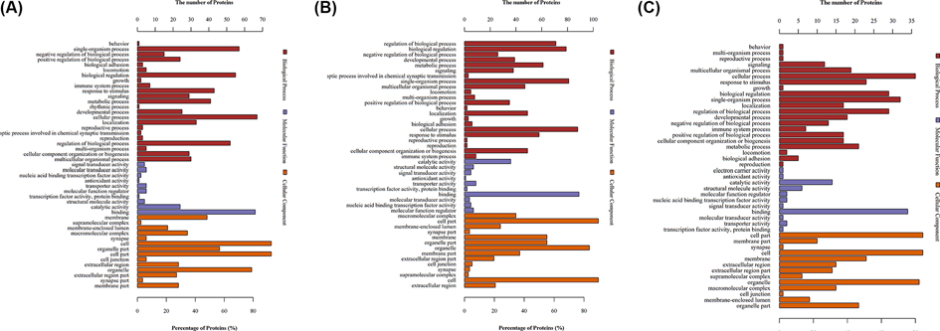
GO analyses of protein functions in (A) M versus C, (B) W versus C and (C) W versus M Gene ontology annotation. The differentially expressed proteins are mainly annotated as protein binding, cell, and single organism process in terms of molecular function, cell composition, and biological process, respectively.

As shown in Figure 3A, in the model group, the proteins with differential levels, as compared with the control groups, were enriched in the biological processes of growth regulation, signal transduction and hormone regulation. Molecular function enrichment was observed in molecular signal transduction, transcription factor activation, protein binding and catalytic reactions. The cellular compositions of the altered proteins were mainly membrane supramolecular complexes and intermembrane macromolecular complexes, which participate in the cell division and intercellular connection (Supplementary Table S3).

As shown in Figure 3B, in the warm acupuncture-treated group, when compared with the control groups, the proteins with altered levels were enriched in the GO biological processes of growth regulation, metabolism process, movement regulation and behavioral regulation. GO molecular function enrichment of differential level proteins was observed in catalytic activity activation, signal transduction activation, antioxidant activity activation, transcription factor activation, protein binding and molecular function regulation. The GO cellular composition of the altered proteins was enriched in intercellular connection of intermembrane, neural synapse and extracellular domains, and the synaptic regulation (Supplementary Table S4).

As shown in Figure 3C, in the warm acupuncture treated group, when compared with the model groups, the proteins with altered levels were enriched in the GO biological processes of signal transduction, regeneration, regulation, growth, immunological reaction and metabolism process; molecular function GO enrichment was observed in nerve conduction electrical carrier activation, antioxidant activity activation, catalytic activation and intermembrane transduction activation. The GO cellular composition of the altered proteins was enriched in cell membrane, synapse, intermembrane tissue, supramolecular complex and intermembrane space, participation in cell junctions (Supplementary Table S5).

### KEGG pathway analysis

**Figure 4:**
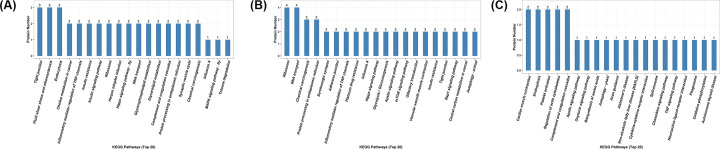
KEGG analyses of protein functions in in (**A**) M versus C, (**B**) W versus C, (**C**) W versus M

As shown in Figure 4A, in the model group, proteins with differential levels, as compared with the control groups, were enriched in the KEGG pathways of tight junctions, atherosclerosis, endocytosis, choline metabolism in cancer, inflammatory mediator regulation of TRP channels, insulin resistance, insulin signal transduction, ribosome, herpes simplex infection, hippocampal signaling, RNA transportation, glycerol phospholipid metabolism, glycerolipid metabolism, complement and coagulation cascades, protein processing in endoplasmic reticulum, synaptic vesicle recycling, chemical carcinogenesis, influenza A and MAPK signaling (Supplementary Table S6).

As shown in Figure 4B, in the warm acupuncture-treated group, when compared with the control groups, proteins with differential levels were enriched in the KEGG pathways of ribosome, RNA transportation, chemical carcinogenesis, protein processing in endoplasmic reticulum, 5-hydroxytryptamine synapse, adhering junctions, inflammatory mediator regulation of TRP channels, platinum resistance, influenza A, Hippo signaling, glycolysis, apelin signaling, mTOR signaling, olfactory transduction, of vascular smooth muscle contraction, insulin resistance, protein binding, Rapl signaling and central carbon metabolism in cancer (Supplementary Table S7).

As shown in Figure 4C, in the warm acupuncture-treated group, when compared with the model groups, the proteins with differential levels were enriched in the KEGG pathways of cardiac muscle contraction, endocytosis, platelet activation, actin cytoskeleton regulation, complement and coagulation cascades, apelin signaling, oxytocin signaling, amino acid biosynthesis, autophagy, axon singling, Alzheimer disease, non-alcoholic fatty liver disease (NAFLD), cytokine–cytokine receptor interaction, spliceosome, chemokine signaling, TNF-α signaling, neuroactive ligand–receptor interaction, phagocytosis, oxidative phosphorylation and autoimmune thyroid disease (Supplementary Table S8).

### Protein expression change

**Figure 5:**
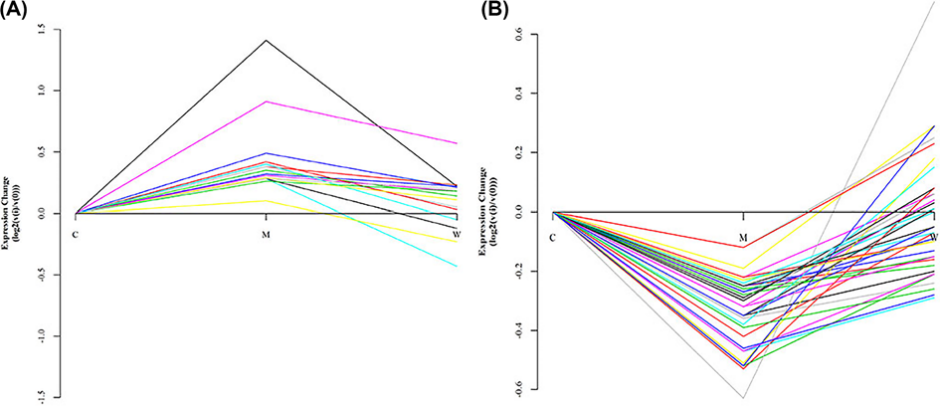
Three groups protein expression change, the warm acupuncture-treated group protein expression verge on control group (**A**) up-regulate protein recovery condition and (**B**) down-regulate protein recovery condition.

As shown in Figure 5, compared with the protein expression change between the three groups, the warm acupuncture-treated group protein expression verge on control group suggests that warm acupuncture treatment is efficacious in improving sleep by regulating the protein expression process in an experimental rat model.

### Enrichment nerve related proteins with differential levels in the warm acupuncture-treated group

In our study, integration of the three groups of clusters analysis, revealed GO and KEGG pathway enrichment for four nerve–related proteins with differential levels in the warm acupuncture treated group, as compared with the model groups. These proteins were Prolargin (PRELP), NMDA receptor synaptonuclear-signaling and neuronal migration factor (NSMF), Transmembrane protein 41B (TMEM41B) and Microtubule-associated protein 1B (MAP1B) (shown in Table 2). These results provided new clues and theoretical basis for the further studies on the treatment mechanism of Mongolian medical warm acupuncture. Moreover, these four proteins were found to be closely related to neuroregulation or insomnia treatment. Among them, prolactin protein prolactin (prelp), which has been shown to be closely related to insomnia treatment, is the focus of our future research.

**Table 2:** Enrichment nerve-related proteins

Gene symbol	Protein name	Function	Fold-change
PRELP	Prolargin	Generation of neurons. Electrical activity and intracellular signal transduction in hypothalamic neurons	1.27
NSMF	NMDA receptor synaptonuclear-signaling and neuronal migration factor	Positive regulation of neuron migration. Regulation of neuronal synaptic plasticity	1.23
TMEM41B	Transmembrane protein 41B	Integral to membrane. nervous system development	1.32
MAP1B	Microtubule-associated protein 1B	Microtubule cytoskeleton organization. Nervous system development	0.48

Proteins that showed increased levels in serum from gastric cancer patients is shown in red and those that showed decreased levels are shown in green.

## Discussion

Insomnia is a subjective experience of sleep and is characterized by a difficulty in falling and/or staying asleep. This condition can result in insufficient sleep quantity to meet an individual’s normal physiological needs, and affects normal life activities [16]. Insomnia is the most common sleep disorder and is associated with high rates of morbidity [17]. However, drug treatment is usually accompanied by many side effects, and with poor long-term efficacy. Long-term administration of hypnotics has been reported to cause problems such as addiction and tolerance, which all together make drug treatment not an ideal therapeutic regimen. Therefore, finding a safe and effective way to treat insomnia will be the focus of future research [18].

Mongolian medical warm acupuncture is one of the most commonly used methods for treatment of insomnia in Mongolian medicine. Mongolian medical warm acupuncture therapy is a traditional Mongolian therapy that prevents and treats diseases by stimulating specific or other sites (acupoints) of the human body, using special silver acupuncture needles, combined with heat stimulation [19,20]. Through integration of the acupuncture and warm effects, and specific responses to acupoint stimulation, Mongolian medical warm acupuncture can treat diseases by regulating multiple aspects of function various body parts, which involves multi-pathway mechanisms and the function of the blood circulatory system, the nervous system and the immune system [21,22].

It functions by unclogging meridians, regulating blood circulation, which results in strengthening the immunity system, and preventing and/or treating diseases [5]. Mongolian medicine annals documentation and modern clinical studies have proven the effectiveness of Mongolian medical warm acupuncture in treating insomnia, and have shown that it is safer than hypnotics as it does not possess any side effects. Thus, treatment of insomnia using Mongolian medical warm acupuncture has been widely accepted by the patients due to its advantages such as high efficiency, safety, no side effects, simplicity and no drug dependence [5,10].

In the present study, the effects of warm acupuncture treat with insomnia rat hypothalamus proteome were investigated, and the data obtained were integrated with related biological information in an attempt to reveal the molecular mechanisms involved in Mongolian medical warm acupuncture for insomnia therapy.

The recent advances in proteomics technologies have provided useful tools for studying the mechanisms of insomnia, which can contribute to the development of insomnia treatments that can be potential applied in clinical practice [23]. In insomnia studies, proteomics has been mainly used to identify physiopathologic mechanisms associated with insomnia and its developing process; however, there is no information on the proteome changes in response to Mongolian medical warm acupuncture. In the present study, iTRAQ-based proteomics analysis was performed to study the proteome changes associated with the application of Mongolian medical warm acupuncture in insomnia treatment. The underlying molecular mechanisms were also investigated by integrating the functional information of the identified proteins, which can potentially provide new or improved treatment strategies in clinical practice.

The experimental insomnia animal model and the quality control were labeled by different iTRAQ labeling reagents in order to detect meaningful changes in proteins, and further bioinformatics analysis was performed to obtain the related biological function of the identified proteins [24].

Gene Ontology (GO) function annotation is a standard function classification system, it provides a set of dynamically updated standardized vocabulary, and describes the characteristics of genes and gene products from three aspects: biological process, molecular function and cellular component [25]. In the present study, we observed GO enrichment in the biological processes of signal transduction, regeneration, regulation, growth, immunological reaction and metabolism process in the warm acupuncture-treated groups [26]. This indicated that warm acupuncture treatment of insomnia involves regulation of nerve signal transduction, growth, and regeneration in rats. We also found GO function enrichment in immune responses, suggesting that Mongolian medical warm acupuncture treated insomnia through up-regulation of immunologic response mechanisms. Furthermore, enrichment was observed in the GO molecular function annotations of nerve conduction electrical carrier activation, antioxidant activity activation, catalytic activation and intermembrane transduction activation, implying that during Mongolian medical warm acupuncture treatment of insomnia rats, nerve signal transduction was stimulated, inter membrane transduction was activated, and the electrical signal conduction was up-regulated. Cellular composition GO analysis revealed enrichment of cell membrane, synapse, intermembrane tissue, supramolecular complex, intermembrane space, and cell junctions, which further indicated differential expression of proteins located in cell membrane and intermembrane space, during Mongolian medical warm acupuncture treatment of insomnia rats. Additionally, enrichment was observed in cell membranes, cell membrane receptor proteins and complicated mesh frame structures composed of signaling polysaccharide macromolecules, which are vital for neurotransmission and signal conduction; thus, cell membrane proteins play vital roles in Mongolian medical warm acupuncture treatment of insomnia. These results also suggested that extracellular matrix proteins are implicated in the initiation and progression of insomnia.

In organisms, proteins function through coordinated interactions with different proteins in order to accomplish a series of biochemical functions [27–29]. Thus, pathway analysis is the most direct and essential method to systematically and comprehensively understand the cellular biological processes involved in the pathogenesis of diseases, and the mechanism of function of medicines [30]. KEGG pathways analysis showed that warm acupuncture treatment groups enriched in oxytocin signaling, amino acid biosynthesis, autophagy, axon singling, cytokine–cytokine receptor interaction, spliceosome, chemokine signaling, TNF-α signaling and neuroactive ligand–receptor interaction. We speculate that the nerve transduction related mechanism was attributed to the coupling of the oxytocin signaling pathway, the neuroactive ligand–receptor interaction and oxidative phosphorylation. Down-regulation of oxytocin protein has been reported to correlate with depression, insomnia and neural regulation. To sum up, KEGG pathway enrichment identified pathways and signaling proteins important for target selections in future studies.

In the present study, integration of the three groups of Cluster analysis, revealed GO and KEGG pathway enrichment for four nerve related proteins with differential levels in the warm acupuncture treated group were PRELP, NSMF, TMEM41B and MAP1B.

PRELP (Prolargin) related to neural regulation, decreased prolactin is associated with depression and insomnia [31]. Prolactin can regulated tuberoinfundibular dopamine neuron discharge pattern, neuronal excitability [32,33]. In the present study, the expression of prolactin increased under warm acupuncture, which is one of the ways to alleviate insomnia. NSMF (NMDA receptor synaptonuclear-signaling and neuronal migration factor) positive regulation of neuron migration and regulation of neuronal synaptic plasticity [34] is crucial for synaptic plasticity and memory, brain-derived neurotrophic factor increases the motility of a particular NMDA /GABA - responsive subset of neural progenitor cells. It is related to the regulation of memory and nerve development [35,36]. Treatment with warm acupuncture can repair neurodevelopmental that is one of the ways to alleviate insomnia. TMEM41B (Transmembrane protein 41B) regulated nervous system development and related to the formation of synapses [37,38]. MAP1B (Microtubule-associated protein 1B) is required for shaping the neural tube, regulated axonal growth and neuronal migration in the central nervous system, and it is highly expressed in nerve injury. Decreased expression after warm acupuncture therapy is one of the manifestations of nerve injury healing [39,40]. These results provided new clues and theoretical basis for the further studies on the treatment mechanism of Mongolian medical warm acupuncture.

## Conclusions

The iTRAQ-based proteomic data analyses presented here elucidate variations of the proteomics involved in the treatment of insomnia by Mongolian medical warm acupuncture. They also increase our understanding of the molecular mechanisms involved in these processes. Distinct protein played an important role in the treatment of insomnia by Mongolian medical warm acupuncture. KEGG and GO analyses enrichment for four nerve–related proteins with differential levels in the warm acupuncture treated group were Prolargin, NMDA receptor synaptonuclear-signaling and neuronal migration factor, transmembrane protein 41B and Microtubule-associated protein 1B to adjust insomnia. It may through regulatory mechanisms of neuronal function on insomnia. It also reveals the potential molecular mechanisms associated with the treatment of insomnia by Mongolian medical warm acupuncture.

## Supplementary Material

Supplementary Tables S1-S8

## Abbreviations

MAP1B: microtubule-associated protein 1B
NAFLD: non-alcoholic fatty liver disease
NSMF: neuronal migration factor
PRELP: prolargin
TMEM41B: transmembrane protein 41B
