# Supplementary material for: iTRAQ-based proteomics analysis on insomnia rats treated with Mongolian medical warm acupuncture
Source: Biosci Rep. 2020 May 7;40(5):BSR20191517. doi: 10.1042/BSR20191517 (PMC7953503; doi:10.1042/BSR20191517)
Supplement: Supplementary Tables S1-S8 [file BSR-2019-1517_supp.zip › BSR-2019-1517.pdf]

1. Supplementary Table S1: Fold change of differentially expressed proteins among C, W and M groups was determined based on the intergroup ratio in iTRAQ reports.

2. Supplementary Table S2: Differentially expressed proteins identified comparisons between M/C, W/C and W/M group protein list.

3. Supplementary Table S3: M/C groups GO functional annotation and analysis

4. Supplementary Table S4: W/C groups GO functional annotation and analysis

5. Supplementary Table S5: W/M groups GO functional annotation and analysis

6. Supplementary Table S6: M/C groups KEGG pathway analysis

7. Supplementary Table S7: W/C groups KEGG pathway analysis

8. Supplementary Table S8: W/M groups KEGG pathway analysis
